# *Eithea lagopaivae*, a new critically endangered species in the previously monotypic genus *Eithea* Ravenna (Amaryllidaceae)

**DOI:** 10.3897/phytokeys.58.13369

**Published:** 2017-08-31

**Authors:** Antonio Campos-Rocha, Alan William Meerow, Edimar Faria Menezes Lopes, João Semir, Juliana Lischka Sampaio Mayer, Julie Henriette Antoinette Dutilh

**Affiliations:** 1 Programa de Pós-Graduação em Biologia Vegetal, Instituto de Biologia, Universidade Estadual de Campinas, Postal Code 6109, 13083-970, Campinas, SP, Brazil; 2 USDA-ARS-SHRS, National Germplasm Repository, 13601 Old Cutler Road, Miami, Florida 33158, USA; 3 Departamento de Biologia Vegetal, Instituto de Biologia, Universidade Estadual de Campinas, Postal Code 6109, 13083-970, Campinas, SP, Brazil

**Keywords:** Anatomy, Asparagales, Endemism, Hippeastreae, São Paulo, Anatomia, Asparagales, Endemismo, Hippeastreae, São Paulo

## Abstract

*Eithea lagopaivae* Campos-Rocha & Dutilh, **sp. nov.** is described as the second species of the formerly monotypic genus *Eithea*. It is characterized by a one flowered inflorescence, completely hollow scape, white or lightly magenta-striated flower that is enclosed by spathe bracts fused for more than the lower fifth of its length. Comments on its range, habitat, phenology, as well as photographs and illustrations are provided. In addition, a distribution map and an identification key for the two species of the genus are presented and anatomical and ecological differences compared. Known by only two small populations exposed to several types of threats and without any guarantee of protection, *E. lagopaivae* is considered a Critically Endangered (CR) species.

## Abstract (pt)

*Eithea lagopaivae* Campos-Rocha & Dutilh, **sp. nov.** é descrita como a segunda espécie do gênero previamente monotípico *Eithea*. Caracteriza-se por apresentar a inflorescência uniflora, escapo totalmente oco, flor alva ou com leves estrias magenta, protegida por brácteas espatáceas fundidas na base por mais de um quinto do seu comprimento. Comentários sobre a sua área de ocorrência, hábitat, dados de fenologia, fotografias e ilustrações são fornecidos. Adicionalmente, um mapa de distribuição e uma chave de identificação para as duas espécies do gênero são apresentados e suas diferenças anatômicas e ecológicas comparadas. Conhecida de apenas duas pequenas populações sujeitas a diversos tipos de ameaça e sem qualquer garantia de proteção, *E. lagopaivae* é considerada uma espécie Criticamente em Perigo (CR) de extinção.

## Introduction

*Eithea* Ravenna (2002) was proposed as a monotypic genus with the transfer of *Griffinia blumenavia* K.Koch & C.D.Bouché ex Carrière, historically a species of somewhat uncertain classification. It was described from material collected in Santa Catarina state, Brazil, cultivated at the Berlin Botanical Garden and originally placed in the genus *Griffinia* Ker Gawl. (Carrière 1867) presumably because of the pseudopetiolate, evergreen leaves. Combinations for both *Hippeastrum* Herb. (Sealy 1937) and *Amaryllis* L. (Traub 1938) were subsequently proposed. Transfer to *Hippeastrum* was indicated due to perceived similarities with the flowers and seeds of *H. reticulatum* Herb. (Sealy 1937) while Traub (1938) proposed the transfer to *Amaryllis*, in line with his belief that the type specimen of *Amaryllis belladonna* L. was an American plant (see Goldblatt 1984, for full details of this controversy). Satô (1938) published a chromosome number of 2*n* = 77 for the species, which would be consistent with *x* = 11 chromosomes as a basic chromosome number for *Hippeastrum* (Naranjo and Andrada 1975, Flory 1977). Almost fifty years later, Traub (1983) proposed the restoration of the species in *Griffinia* based on the work of Arroyo (1982), who reported 2*n* = 20 chromosomes for the species.

A phylogenetic analysis of nrDNA ITS sequences resolved *G. blumenavia* as having a closer relationship to the genus *Rhodophiala* Presl, than with either *Griffinia* or *Hippeastrum* (Meerow et al. 2000). This work also reported a chromosome number of 2*n* = 18 for *G. blumenavia*, the number found in most *Rhodophiala* species (Satô 1942, Ficker 1951, Naranjo 1969, Flory 1977). Meerow et al. (2000) considered that these findings would justify the separation of *G. blumenavia* from *Griffinia* and *Hippeastrum* with recognition as a distinct monotypic genus. The authors of the current paper analyzed different individuals of the species and found the main somatic chromosome number of 2*n* = 18. However, a few cells from some individuals exhibited 19 to 20 chromosomes, perhaps explaining Arroyo (1982) report of 2*n* = 20. These additional smaller or supernumerary chromosomes are considered B-chromosomes (Jones and Rees 1982, Dutilh 1989, Ising 1990, Ising and Wide-Andersson 1991) due to their erratic behavior. Ravenna (2002) described the species as *Eithea blumenavia* (K.Koch & C.D.Bouché ex Carrière) Ravenna based on some morphological considerations, not on phylogeny nor chromosome number.

Currently, *Eithea* is positioned in tribe Hippeastreae, which includes *Hippeastrum* and *Rhodophiala* (Meerow et al. 2000, Oliveira 2012, García et al. 2014, 2017). In the same studies, *Griffinia*, sister of monotypic *Worsleya*, is included in the tribe Griffinieae Ravenna, a strongly supported clade. *Eithea* has some unusual morphological attributes for Hippeastreae, such as pseudopetiolate oblanceolate leaves with attenuate base, as well as globose seeds with elaiosome, features also found in *Griffinia*. However, testa of *Eithea* seeds contains phytomelanin, a typical trait of Hippeastreae, not found in *Griffinia*.

At the end of the 1990s, the researcher Celso do Lago Paiva discovered the new species in Piracicaba, São Paulo state, Brazil. Morphological, ecological and anatomical analyses conducted on material of the species over the past ten years have confirmed that it is an undescribed species of *Eithea*.

## Material and methods

The following national and international herbaria with the most important collections of Brazilian Amaryllidaceae, particularly those from the state of São Paulo, were visited: BR, C, ESA, HRCB, IAC, K, MBM, MO, NY, RB, SP, SPF, SPSF, UEC, and US (acronyms according to Thiers 2017). The terminology used for the morphological description follows Radford et al. (1974), Meerow and Snijman (1998), and Stearn (2004), with minor modifications. In addition, comments on the conservation status of the taxon are provided as recommended by IUCN (2016). GeoCAT (Bachman et al. 2011) was used to calculate Area of ​​Occurrence (AOO) and Extent of Occurrence (EOO). Climatic data was obtained from Banco de Dados Climáticos do Brasil (EMBRAPA 2003) for the municipalities of Indaial (Santa Catarina) and Piracicaba (São Paulo). These two localities present collections of *E. blumenavia* and *E. lagopaivae* respectively. The map was drawn with ArcGIS 10.5 (ESRI).

For the morphological analysis, measurements were made on at least 15 individuals of each species, fresh plants and exsiccates. For the anatomical analysis, slides were prepared with fresh mature leaves of *E. blumenavia* and *E. lagopaivae*. At least five fully expanded and mature leaves of each species were fixed in Karnovsky (Karnovsky 1965) for 24 h, dehydrated in ethanol series (10%, 30%, 50% - 1 h each) and stored in 70% ethanol. Samples of leaves from both species were selected, encompassing the middle region of the pseudopetiole and the lamina, which was subdivided into midrib region and area between the midrib and margin. Infiltration was performed in plastic resin (Leica Historesin®) according to manufacturer’s instructions. Transverse and longitudinal sections of 7 µm thickness were obtained with manual rotary microtome (Leica®) and stained with 0.05% toluidine blue (Sakai 1973) in citrate buffer. Slides were mounted in synthetic resin “Entellan®” (Merck®) and images were obtained with an Olympus DP71 digital camera attached to an Olympus BX51 microscope.

Vouchers of the species and populations were deposited at UEC.

## Results and discussion

### Eithea lagopaivae

Taxon classificationPlantaeAsparagalesAmaryllidaceae

Campos-Rocha & Dutilh sp. nov.

urn:lsid:ipni.org:names:77165357-1

Figures 1 , 2

#### Diagnosis.

*Eithea lagopaivae* differs from *E. blumenavia* (Figure 3) by its smaller size, one flowered inflorescence (vs. 2–6, very rarely 1), a fully hollow scape (vs. solid in the lower fifth), terminated by spathe bracts fused for more than the lower fifth of their length (vs. free or fused up to the lower fifth), absence of bracteoles (vs. presence), white or only very lightly striated flowers (vs. strongly striated) and lateral and lower petals of similar width (vs. lateral petals up to twice the width of the lower).

#### Type.

BRAZIL. São Paulo: Piracicaba, sub-bosque de uma plantação comercial de *Eucalyptus* abandonada, 07 Dec 2016, *A. Campos-Rocha 1654* (holotype: UEC!; isotypes: NA!, RB!).

#### Description.

Geophytic herb 12–25 cm tall. Bulb subterranean, globose to ovoid or obovoid, tunicate, whitish or with a thin grey-brownish outer tunic, 1.3–3.5 cm long and 1.2–3 cm diameter; neck formed by sheathing leaf bases up to 4.5 cm long and 3–8 mm diameter (occasionally very short to absent). Leaves 1–3(–4), suberect, dark green adaxially, pale green abaxially, frequently pseudopetiolate; pseudopetiole flattened adaxially, rounded abaxially, greenish, with reddish pigmentation near the base or throughout its length, up to 9.5 cm long, 2–5.5 mm wide; lamina linear, narrowly elliptic or oblanceolate to slightly falcate, apex acute, frequently asymmetric, base attenuate, margin flat, venation transverse reticulate (with short transverse veins between the longitudinal ones), midrib inconspicuous adaxially, prominent abaxially, 8–20.8 × 1.1–2.6 cm. Inflorescence one flowered; scape erect, cylindrical, slightly laterally compressed, hollow and fragile, greenish, sometimes with reddish pigmentation near the base, 7.8–30 cm long and 2.4–6 mm diameter, elongating and becoming decumbent with fructification; spathe bracts 2, tubular, fused in the basal 0.4–2.4 cm, apex acute, whitish, generally light rose colored at the tip before opening, turning papery, 1.7–3.8 cm long. Pedicels greenish, (0.3–)1.3–5.5 cm long, often elongating with fructification to 6.5 cm long. Perigone campanulate to infundibular, white (in bud white with a rose colored tip), usually with faint thin magenta striations on the sepals and petals, especially on the upper sepal, with greenish pigmentation near the base, mostly close to the midrib, 3–5.8 cm long; hypanthium greenish, 2–4.5 mm long, paraperigone of fimbriae 0.5–2 mm long at the throat. Sepals much wider than the petals, oblanceolate to obovate, the upper one wider and longer, apex acute, apicule subapical; upper 2.7–5.6 × 1–2.4 cm, apicule 0.8–2 mm long; lateral 2.5–5.4 × 0.6–2 cm, apicule 0.6–1.4 mm long. Petals oblanceolate, apicule inconspicuous or absent; lateral 2.5–5.5 × 0.6–1.4 cm; lower slightly narrower, 2.5–5.5 × 0.4–1.2 cm. Filaments 6, in four different lengths, inserted at the mouth of the hypanthium tube, shorter than limb segments, declinate-ascending, free portion white; upper episepal 1.1–2.5 cm long; lateral episepal 1–2.2 cm long; lateral epipetal 1.7–4 cm long; lower epipetal 1.6–3.8 cm long. Anthers oblong to oblong-reniform, dorsifixed, versatile, dehiscing longitudinally, 2.5–5 mm long before anthesis; pollen pale yellow. Ovary trilocular, obtuse trigonal, obovoid, greenish, 3.5–9 mm long and 3–8 mm diameter; 8–14 ovules per locule; ovules 0.6–1 mm long. Style declinate-ascending, white, occasionally with greenish pigmentation near the base, 2.6–5 cm long; stigma trifid, white, lobes already expanded when the flower opens, occasionally of different lengths, 1.5–4.5 mm long. Fruit capsule loculicidal, globose to globose-compressed trilobed, greenish when ripe, occasionally with reddish pigmentation, cream colored inner side, 1–2.5 cm long and 1.2–2.6 cm diameter. Seeds irregular, angular, with grey brownish to black testa containing phytomelanin, 3.5–6 mm long and 3–5.5 mm diameter, with wrinkled elaiosome up to 4.5 mm long.

#### Distribution, habitat and ecology.

*Eithea lagopaivae* is known from only two small populations separated about 50 km, each composed of less than 50 individuals. The type population (Piracicaba) occurs in the understory of an abandoned *Eucalyptus* plantation, next to fragments of deciduous and semideciduous forest, where the Corumbataí river meets the Piracicaba river. The second is located in a small fragment of semideciduous forest, near the junction of the basins of the Piracicaba and Tietê rivers in the municipality of Tietê (Figure 4). Both fragments are located on gravelly soils of litholic limestone origin (Oliveira and Prado 1989). The region presents a well-defined seasonality, with total annual rainfall of 1230 mm and precipitation of 50 mm or less, for six months, during autumn and winter. During spring and summer, rainfall exceeds 100 mm for six months, reaching close to 250 mm in January (EMBRAPA 2003). Ants were observed removing elaiosomes from the seeds of *E. lagopaivae* in their natural habitat, indicating that these animals might be dispersal agents, as is known for *Griffinia*.

#### Phenology.

*Eithea lagopaivae* has been collected in bloom between October and January, and occasional blooming occurs until early March. Fruits have been observed from November.

#### Conservation status.

With estimated AOO of 8 km^2^ and EOO of 13.7 km^2^, *Eithea lagopaivae* can be considered as Critically Endangered [CR, B1ab(iii) + B2ab(iii)], due to the low number of known locations (≤ 5) and decline in quality of habitat (IUCN 2016). In the municipality of Piracicaba, at the end of the year 2016, when the species was again visited, two small scattered groups were encountered ca. 500 meters apart. The smaller of the two groups was in a trash dump on the side of the wooded area, and the second in an area of ​​higher humidity, near a small stream. This fragment, on the edge of the urban sprawl of Piracicaba, is highly disturbed and subject to regular episodes of fire. The population of the Tietê municipality is in a slightly larger fragment of semideciduous forest with an impoverished understory, intense edge effects, with many lianas and invasive exotics. The area is located within a livestock breeding facility.

#### Etymology.

The epithet is a tribute to Celso do Lago Paiva, environmental analyst at ICMBio, who has collected the plant for the first time and has dedicated his life to the study and conservation of the flora of Brazil.

#### Additional specimens examined.

BRAZIL. São Paulo: Piracicaba, 18 Mar 1999, *J. Dutilh* s.n. (UEC-170468!); 17 Nov 1999, *J. Dutilh* s.n. (UEC-174104!); 29 Nov 1999, *J. Dutilh* s.n. (UEC-174105!); em cultivo no Jardim Botânico Plantarum, Nova Odessa-SP, 10 Oct 2012, *A. Campos-Rocha 810* (NA!, RB!, UEC!); em cultivo em Campinas-SP, 10 Oct 2013, *A. Campos-Rocha* & *J. Dutilh 1165* (NA!, UEC!); plantação abandonada de *Eucalyptus*, 09 Oct 2016, *A. Campos-Rocha* & *R.M. Goffi 1626* (UEC!); plantação de *Eucalyptus* abandonada, 20 Nov 2016, *A. Campos-Rocha 1647* (NA!, UEC!). Tietê, 20 Nov 2001, *J. Dutilh* & *L.C. Bernacci* s.n. (UEC-170469!); *L.C. Bernacci et al. 4483*, fragmento de floresta semidecídua, 03 Mar 2017 (IAC!, UEC!).

#### Notes.

*Eithea lagopaivae* and *E. blumenavia* form a clade with maximal support in all phylogenetic analyses performed by García et al. (2017). *Eithea lagopaivae* can be distinguished from *E. blumenavia* by a number of characteristics (Table 1). It is a smaller plant (ca. 12–25 cm), usually with 2-3 leaves, rarely 4, which are deciduous before the onset of winter. *Eithea blumenavia* however is evergreen, with 2-8 leaves, and up to 50 cm in height, although specimens of extremely reduced size are known, also with several flowers.

*Eithea lagopaivae* is known from an area originally of deciduous and semideciduous forests with a well-defined dry season. In turn, *E. blumenavia* is found in wetlands of the Atlantic rainforest, from the south of the state of São Paulo to eastern Santa Catarina (Dutilh 2010, Dutilh and Oliveira 2015) (Figure 4), especially in the coastal mountains. The region has some of the highest average annual rainfall (1650 mm) of any area of extra-Amazonian Brazil, distributed throughout the year, but more intensely during the summer, although with an average under 200 mm/month. From April to July, monthly averages are close to 100 mm (EMBRAPA 2003).

*Eithea blumenavia* is considered an Endangered (EN) species (MMA 2014).

#### Anatomy.

The three most obvious anatomical characteristics differentiating the two species of *Eithea* are: 1. Margins and cortex of the pseudopetiole (Figure 5A–B); 2. Ornamentation and shape of the epidermal cells on the adaxial side of the leaf blades (Figure 5C–D); 3. Presence or absence of protrusions on the upper side of the blades in the region of the midrib (Figure 5I–J).

Cross section of pseudopetiole margins of *E. lagopaivae* are flatter, more laminar (Figure 5A, arrow) than those of *E. blumenavia*, which are angular (Figure 5B, arrow). The pseudopetiole is composed by chlorenchyma, aerenchyma and vascular bundles. In *E. lagopaivae*, 1–3 aerenchyma lacunae were found below the vascular bundles (Figure 5A), while in *E. blumenavia* several lacunae above and below the bundles could be observed (Figure 5B).

In the cross section of leaf lamina, epidermal cells were more elongated in *E. lagopaivae* and polyhedral in *E. blumenavia* (Figure 5C–D, respectively). We found periclinal thickening on the outer wall of the epidermal cells of both species as occurs in *Hippeastrum puniceum* (Lam.) Kuntze (Alves-Araújo et al. 2012). Ornamentation of the external periclinal epidermal cell wall of *E. blumenavia* (Figure 5D, arrow) was not found in *E. lagopaivae* (Figure 5C). Mesophyll of *E. lagopaivae* and *E. blumenavia* is composed of about 6–8 layers of chlorenchyma with arm-palisade cells (also called arm-cells, H-palisade or H-cells) (Figure 5E–F), which showed their typical morphology in paradermic sections (Figure 5G–H). Arm-cells were first described by Haberlandt (1880) as a morphological modification of palisade cells and seem to be more common in plants of forest understory, probably increasing photosynthetic capacity (Chatelet et al. 2013). In the midrib region, the lacunae of the aerenchyma were larger and wider in *E. lagopaivae* than in *E. blumenavia* (Figure 5I–J).

A protrusion on the abaxial leaf surface opposite the central vascular bundle was evidenced in both species (Figure 5I–J, arrows) and the parenchyma cells in this region were regular and rounded. However, adaxial surface of the leaf in *E. lagopaivae* was flat (Figure 5I), while in *E. blumenavia* it was possible to observe two protrusions opposite to the vascular bundles adjacent to the midrib (Figure 5J, arrowheads). The alternation of aerenchyma with vascular bundles found in *Eithea* species was described for other species of the family (Arroyo and Cutler 1984, Meerow 1989, Raymúndez et al. 2000, Alves-Araújo et al. 2012). In *Eithea*, lacunae of aerenchyma in the pseudopetiole and leaf blade may have been originated by lysis, as suggested for *Griffinia*, *Habranthus*, *Hippeastrum* and *Nothoscordum* Kunth (Alves-Araújo et al. 2012); however leaf development studies are needed to confirm this hypotheses.

**Figure 1.**
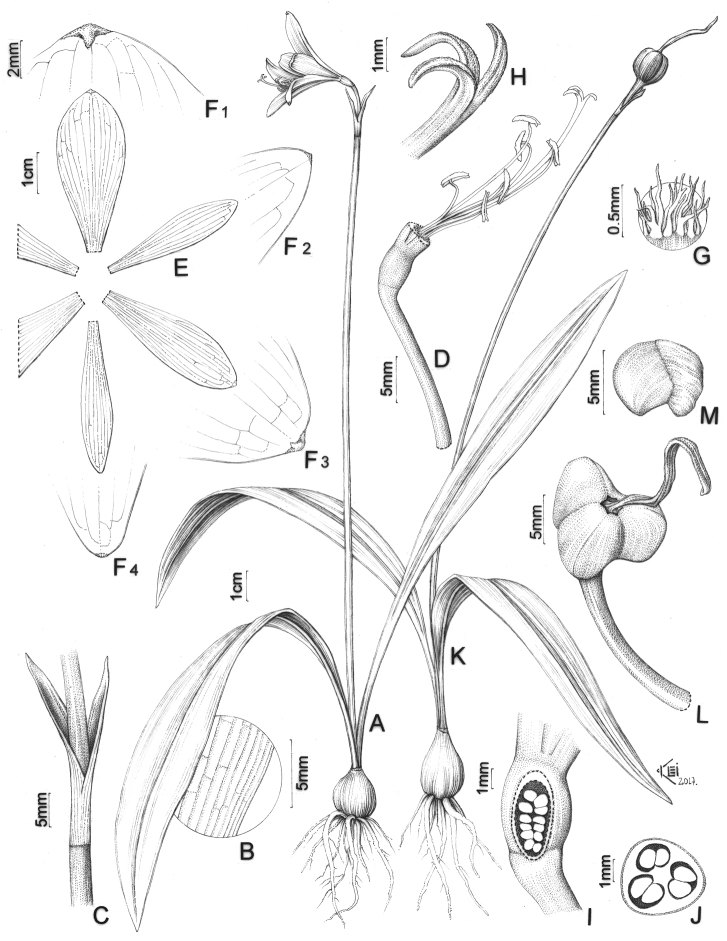
*Eithea lagopaivae* **A** Habit in flower **B** Detail of leaf venation **C** Spathe bracts **D** Flower with perigone removed, showing stamens and style **E** Sepals and petals **F** Tips of sepals and petals **F1** Upper sepal **F2** Lateral petal **F3** Lateral sepal **F4** Lower petal **G** Detail of fimbriae of the paraperigone **H** Stigma **I** Longitudinal section of the ovary **J** Cross section of the ovary **K** Habit in fruit **L** Fruit **M** Seed.

**Figure 2.**
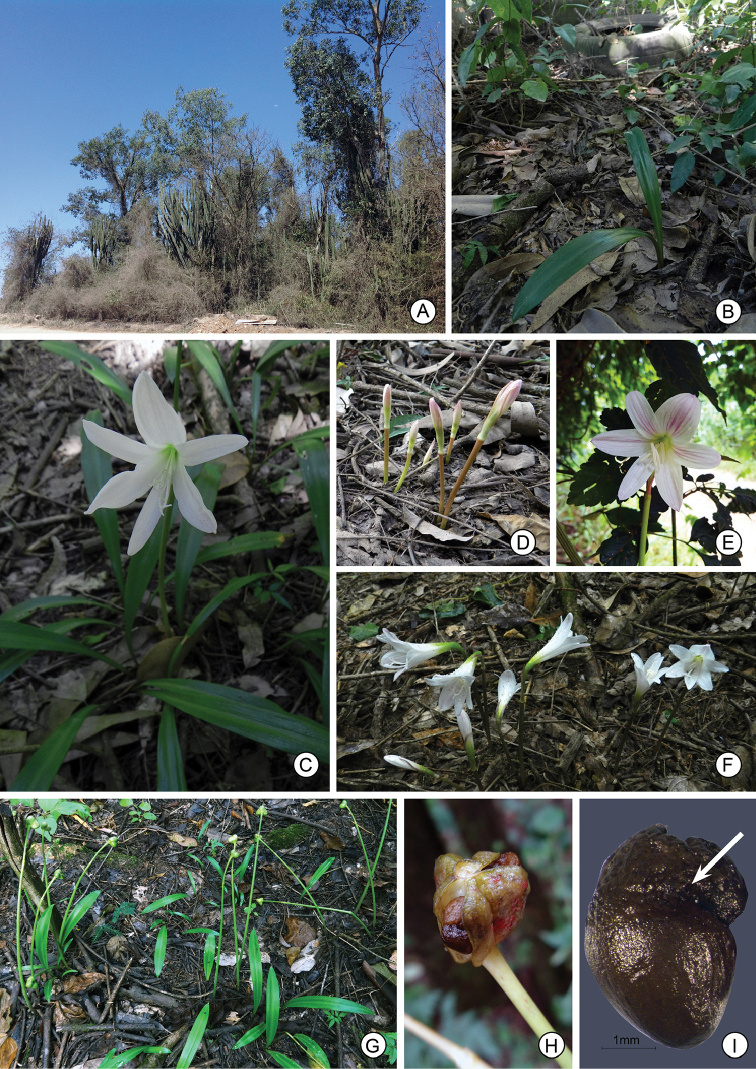
*Eithea lagopaivae* **A** Typical habitat (October 2016) **B** Individual plant flowering amid trash dumped at type locality **C** Flowering plant (*Campos-Rocha 1647*) **D** Flower buds **E** Flower, frontal view (*Bernacci 4483*) **F** Flower buds and flowers (*Campos-Rocha 1654*) **G** Plants in fruit **H** Mature capsule exposing the seeds **I** Seed (elaiosome indicated by the arrow).

**Figure 3.**
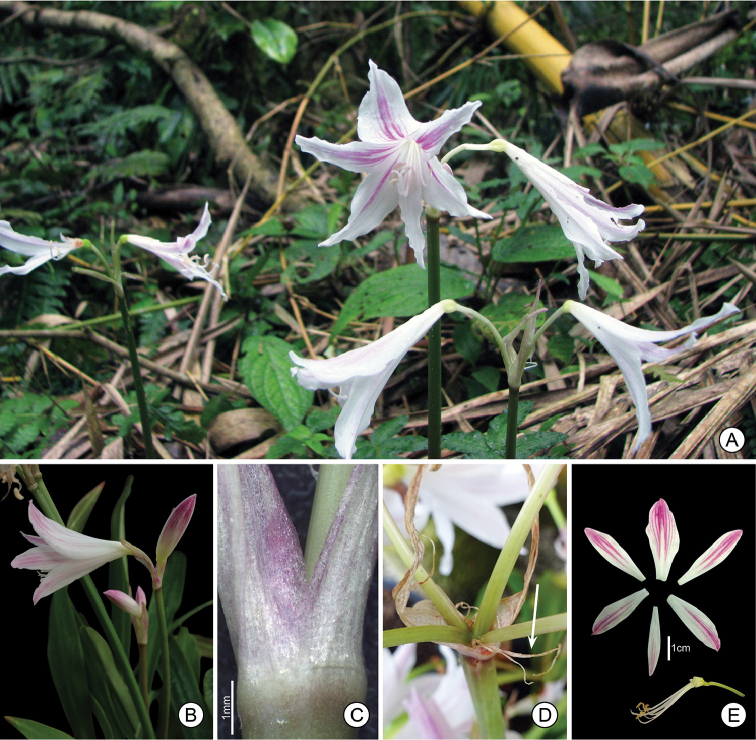
*Eithea blumenavia* **A** Flowering plants in habitat **B** Lateral view of inflorescence **C** Detail of spathe bracts **D** Bracteoles (white arrow) **E** Sepals and petals (adaxial view) separated from stamens and style. **B–E** of *Campos-Rocha 1624*, UEC.

**Figure 4.**
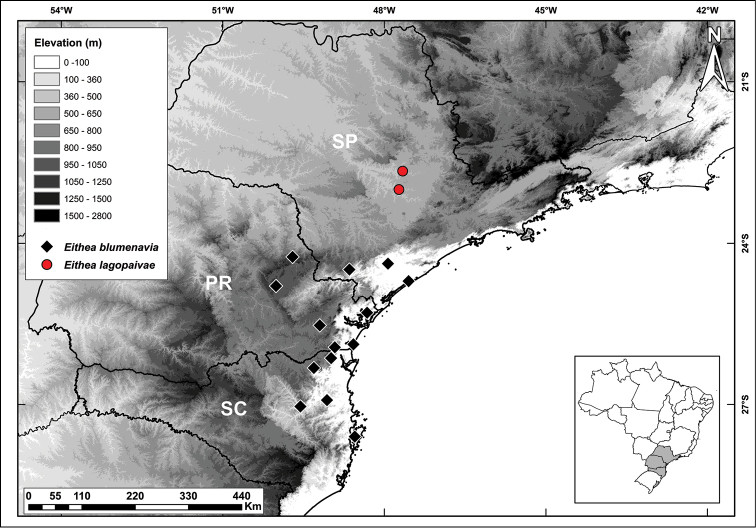
Distribution map showing collections of *Eithea lagopaivae* (red circles) and *E. blumenavia* (black diamonds). PR = Paraná. SC = Santa Catarina. SP = São Paulo.

**Table 1.** Ecological, morphological and anatomic character states that distinguish *Eithea lagopaivae* from *E. blumenavia*. CS = cross section.

Character state	*Eithea lagopaivae*	*Eithea blumenavia*
Habitat	Semideciduous and deciduous forest	Rain forest
Foliage	Deciduous	Perennial, rarely deciduous
Scape	Fully hollow	Solid in lower fifth
Spathe bracts	Fused more than 1/5^th^ basally	Free to fused for up to 1/5^th^ basally
Bracteoles	Absent	Present
No. flowers per inflorescence	1	2–6, rarely 1
Perigone color	White, sometimes with a few magenta striations	White with many conspicuous, magenta striations
Width ratio of lateral to lower petals	4:4 to 4:3	4:3 to 4:2
Pseudopetiole margins	Laminar	Angular
Adaxial epiderm cells (leaf blade) in CS	Elongated rectangular	Polyhedral
Ornamentation of external periclinal epidermal cell wall (leaf blade)	Absent	Present
Adaxial surface in the midrib region (leaf blade) in CS	Flat	2 protrusions

**Figure 5.**
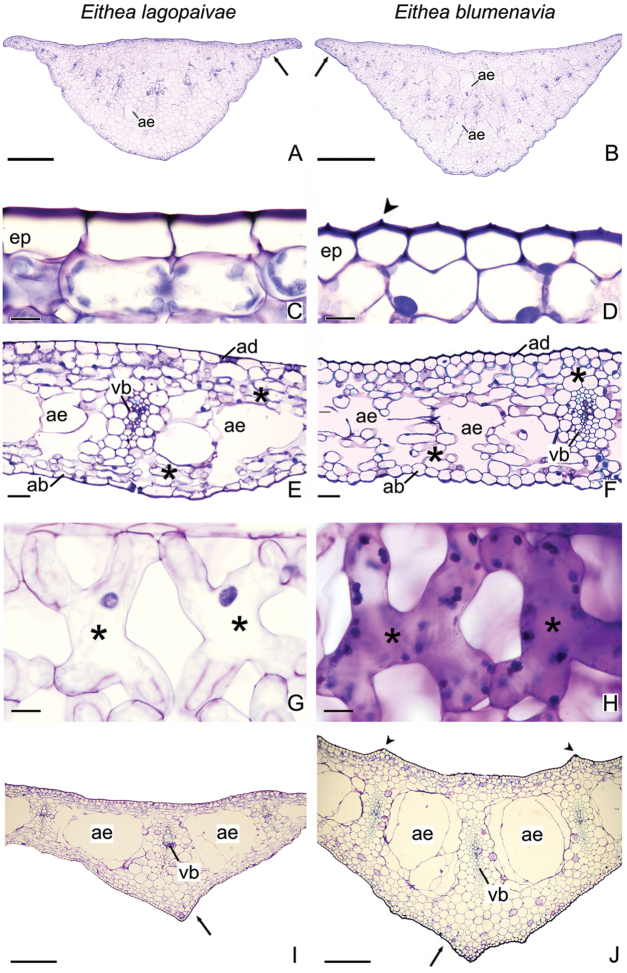
Comparative leaf anatomy of *Eithea lagopaivae* and *E. blumenavia*. **A** Cross section of the pseudopetiole of *E. lagopaivae* and **B** *E. blumenavia* **C** Cross section detail of the adaxial epidermis of *E. lagopaivae* and **D** *E. blumenavia* **E** Cross section of the leaf blade of *E. lagopaivae* and **F** *E. blumenavia* **G** Longitudinal paradermic section detail of the leaf blade, showing the arm-cells of the chlorenchyma in *E. lagopaivae* and **H** *E. blumenavia* (slide of stained fresh material) **I** Cross section of the leaf blade in the midrib region of *E. lagopaivae* and **J** *E. blumenavia*. * = arm-cell; ab = abaxial epidermis; ad = adaxial epidermis; ae = aerenchyma; ep = epidermis; vb = vascular bundle. Scales bars: 500 µm (**A, B**); 10 µm (**C, D**), 50 µm (**E, F**), 20 µm (**G, H**); 200 µm (**I, J**).

### Key to the species of *Eithea*

**Table:** 

1	Inflorescence one flowered; bracts fused for more than the lower 1/5th; scape completely hollow; flowers white or only with a few narrow magenta striations; ratio between the width of the lateral and lower petals 4:3 to 4:4; plants from deciduous and semideciduous forest	***E. lagopaivae***
–	Inflorescence with 2–6 flowers, very rarely 1; bracts free or fused up to the lower 1/5th; scape solid towards the base; flowers with many conspicuous magenta striations; ratio between the width of the lateral and lower petals 4:2 to 4:3; plants from rainforest	***E. blumenavia***

## Supplementary Material

XML Treatment for Eithea lagopaivae
